# RESECTION OF ANKLE TUMOR LESION AND RECONSTRUCTION WITH THE USE OF ALLOGRAFT

**DOI:** 10.1590/1413-785220233105e266018

**Published:** 2023-10-23

**Authors:** DIEGO PEREZ DA MOTTA, BEATRIZ GOMES ARRUDA, RAFAEL DE CASTRO E SILVA PINHEIRO, GABRIEL ARAÚJO RIBEIRO, BRUNA CANTERI DELOCCO, BRUNO DE OLIVEIRA FIORELLI, EDUARDO ALESSANDRO LIMA WITTE, WALTER MEOHAS

**Affiliations:** 1 Fellow in Oncological Orthopedics, Instituto Nacional de Traumatologia e Ortopedia Jamil Haddad, Rio de Janeiro, RJ, Brazil; 2 Orthopedic Resident, Instituto Nacional de Traumatologia e Ortopedia Jamil Haddad, Rio de Janeiro, RJ, Brazil; 3 Instituto Nacional de Traumatologia e Ortopedia Jamil Haddad, Centro de Atenção Especializada em Ortopedia Oncológica, Rio de Janeiro, RJ, Brazil

**Keywords:** Bone Neoplasms, Bone Nails, Tibia, Orthopedic Procedures, Neoplasias Ósseas, Pinos Ortopédicos, Tíbia, Procedimentos Ortopédicos

## Abstract

Reconstruction of the distal third of the tibia due to resection of a malignant tumor has some hindering factors, such as a thin subcutaneous layer, neurovascular bundles that cross compartments, prolonged operative duration, specific orthopedic material, and a trained multidisciplinary team. Allografting with material from tissue banks is part of this orthopaedic arsenal. Objective: To describe the protocol used at Instituto Nacional de Traumatologia e Ortopedia Jamil Haddad. Methods: Series of six cases subjected to resection with oncologic margins, allograft reconstruction, and use of a retrograde ankle nail as limb-salvage surgery. Three of the six patients were women, the lesions were on average 9.3 cm long, and the average operative duration was 3.25 hours. Results: The main short-term complication (≤ 30 days) was peroneal nerve palsy, while the main long-term complication (> 30 days) was surgical site infection (two cases). Consolidation of the two foci occurred in three patients, and two patients developed asymptomatic pseudoarthrosis of the proximal focus with consolidation of the distal focus. Conclusion: Despite the complications, the proposed surgery gives patients the chance to preserve their limb in the face of immediate radical surgery. *Level of Evidence IV, Case Series*.

## RESUMO

A reconstrução do terço distal da tíbia devido à ressecção de tumor maligno apresenta alguns fatores que dificultam sua realização, como camada subcutânea delgada, feixes neurovasculares que transpassam os compartimentos, tempo cirúrgico prolongado, material ortopédico específico e equipe multidisciplinar treinada. O aloenxerto de banco de tecido faz parte deste arsenal ortopédico. Objetivo: Descrever o protocolo realizado no Instituto Nacional de Traumatologia e Ortopedia Jamil Haddad. Métodos: Série de seis casos submetidos à ressecção com margens oncológicas, reconstrução com aloenxerto e uso de haste retrógrada de tornozelo como cirurgia preservadora do membro. Três dos seis pacientes eram do sexo feminino, as lesões tinham em média 9,3 cm de comprimento e o tempo cirúrgico médio foi de 3,25 horas. Resultados: A principal complicação de curto prazo (≤ 30 dias) foi a paralisia do nervo fibular, enquanto a principal complicação de longo prazo (> 30 dias) foi a infecção do sítio cirúrgico (dois casos). A consolidação dos dois focos ocorreu em três pacientes, e dois pacientes evoluíram para pseudoartrose assintomática do foco proximal com consolidação do foco distal. Conclusão: Apesar das complicações, a cirurgia proposta permite ao paciente a chance de preservar seu membro diante de uma cirurgia radical imediata. *Nível de Evidência IV, Série de Casos*.

## INTRODUCTION

Malignant musculoskeletal tumors in the foot and ankle are uncommon and account for about 1% to 5% of all tumor lesions.^1^ Most of these lesions derive from muscle tissue and have a benign behavior, while malignant lesions are rare, although often underestimated.^2^

A study performed at Universidade de Coimbra showed that 56% of cases of musculoskeletal tumors of the foot and ankle occur in women and 44% in men, aged 15 to 76 years old. Most lesions (78%) were benign. In terms of location, 88% were soft tissue tumors, 12% were bone lesions, and the most frequent histological diagnosis was giant cell tumor.^3^ The ratio of benign neoplasms in this region is over 5:1 in relation to malignant neoplasms.^4^

The symptoms of neoplasms of the foot and ankle are diverse and vary depending on the patient’s type of lesion. According to the anatomy of the region, little subcutaneous and muscle tissue covers the anteromedial region (mainly), making the lesions easily palpable. Another factor is the proximity of the anatomical compartments of the leg through which the neurovascular bundles that cross the joint pass, causing nonspecific complaints.

The anatomy of the leg has two bones that constitute the bony structure (tibia and fibula) and is divided into three compartments (anterior, lateral, and posterior, which is subdivided into superficial and deep posterior). These compartments include the muscles and neurovascular bundles. The path of these bundles is not linear, and they can pass from one compartment to another. Due to the proximity of compartments, the size of the subcutaneous layer, and the crossing of structures through the compartments, lesions located in the distal portion of the tibia generally present symptoms early and are easy to palpate. Expansive lesions that involve more than one compartment can lead to loss of function and motor/sensory alterations in the nerves of different compartments.^5^ The compaction of the compartments in this region and the transposition of neurovascular bundles can make complaints inaccurate. Due to the proximity of anatomical structures, the lesions are generally palpable or symptomatic from the disease onset, facilitating early diagnosis.^5^

Ankle tumors have low incidence and anatomical, clinical, and histological particularities that imply poorly elucidated therapeutic proposals, mostly of surgical nature.^6^ Traditionally, transtibial amputation is chosen for malignant lesions, since the main obstacles to limb-salvage surgery are the small amount of soft tissue coverage at the site and the difficulty in obtaining an adequate resection margin.^1^

Reconstruction and wide resection surgeries associated or not with arthrodesis and allogeneic or autologous bone graft with vascularized fibula require a trained surgeon for a positive result.^7^ The tumor must be resected with an adequate margin, preserving the adjacent tendons and neurovascular structures, which are essential for maintaining limb functionality.

Another therapeutic possibility described in the literature is ankle replacement, although its indication is restricted.^8^ However, this procedure is associated with complications such as cement loosening, arthroplasty and muscle failure, which can be avoided by opting for arthrodesis treatment.^9^

This study aims to show the resection of the tibial distal portion with wide margins, reconstruction with an allograft from the tissue bank of Instituto Nacional de Traumatologia e Ortopedia Jamil Haddad (INTO), and the use of a retrograde ankle nail for fixation, evaluating cases subjected to proposed surgery from January 2012 to May 2022.

## METHODS

This study was approved by the INTO Research Ethics Committee, under CAAE 56177822.0.000.5273. Six cases of malignant bone tumors in the ankle treated by allograft arthrodesis from January 2012 to May 2022 were evaluated.

Patients with a diagnosis of malignant bone tumor in the ankle with indication for wide surgical resection involving only the distal region of the tibia and its articular face with the talus were included.

The exclusion criteria were: (1) benign bone tumors; (2) extensive lesions that affect the vascularization and do not allow limb-salvage surgery; (3) patients who do not agree with the terms of the study; and (4) patients who did not sign the informed consent form.

Data were collected using a physical record and imaging tests (radiography, computed tomography, and nuclear magnetic resonance). The following information was considered: sex, age, operative duration, number of bags transfused in the postoperative period, comorbidities, ASA anesthesia classification, medical evolution, date of hospitalization, date of surgery, date of hospital discharge, surgical description, and anatomopathological reports.

### Surgical technique

#### Preoperative period

To be cleared for surgery, patients are assessed for clinical and soft tissue condition and the distance between the line of the ankle joint and the uninjured area, proximal to the bone lesion, where no imaging scan shows it, is measured in centimeters. This planning is important for the request to reserve the allograft in the tissue bank.

#### Intraoperative period

**Figure 1:**
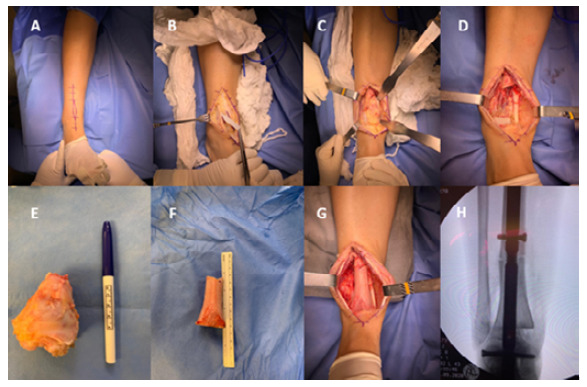
Surgical steps: A) Marked surgical access, covering the biopsy scar; B) Proximity to the peroneal nerve; C) Bone lesion; D) Bone gap after removal of the lesion; E) Bone lesion; F) Prepared allograft; G) Grafted gap; H) Fluoroscopy with synthesis.

For the technique used, patients are placed in the supine position under anesthesia using a pneumatic cuff at 300 mmHg at the limb root to be operated. Surgical access is made in the anteromedial region of the distal portion of the leg, extending proximally to the area to be resected up to the topography of the Chopart joint, with the scar from the previously performed biopsy (Figure 1A).

Soft tissue dissection follows oncological principles, aiming at wide margins free of tumor lesions (Figures 1B and 1C).

In the intraoperative period, the previously determined distance is measured with an sterile ruler, adding 2 to 4 cm proximally to obtain free surgical margins. Diaphyseal osteotomy of the tibia is performed with an oscillating saw attached to the motor. Subchondral osteotomy of the proximal articular portion of the talus continues, minimizing the risk of pseudoarthrosis. Osteotomy of the distal portion of the ipsilateral fibula is unnecessary (Figure 1D).

The osteotomized specimen removed en bloc has its longitudinal measurement estimated and is sent for histopathological analysis in a suitable container with formaldehyde solution.

The bone defect produced is then measured for the suitability and preparation of the allograft to be used. Preference is given to allografts from the distal region of the tibia and the diaphysis of the femur, due to their more compatible diameter and length (Figures 1E and 1F).

The allograft is then placed in the bone defect created and tibiotalocalcaneal arthrodesis is performed with a retrograde ankle nail using an olive guide wire (Figure 1G). The nail is blocked proximal to the allograft (in the patient’s tibia), as well as in the hindfoot region (Figure 1H).

At the end, the pneumatic cuff is deflated, hemostasis is checked, and suturing is performed in reverse planes. Patients are released with a pressure dressing.

#### Postoperative period

Patients are discharged with a removable walking boot and are instructed not to apply any load to the operated limb for six weeks. The use of crutches is therefore recommended.

**Figure 2:**
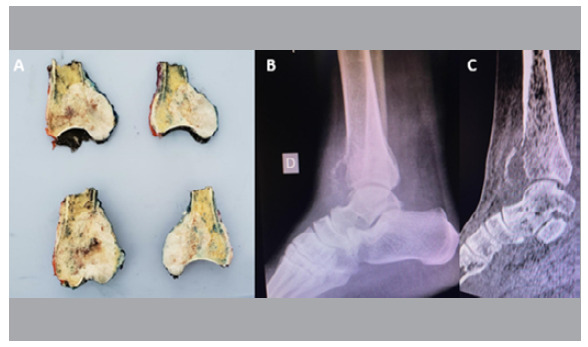
A) Anatomical specimen; B) Lateral X-ray of the ankle; C) Sagittal section of a computed tomography of the ankle.

Outpatient follow-up visits are paid weekly until the surgical stitches are removed. After that, patients are monitored monthly with X-rays to assess bone healing at the healthy bone/allograft interfaces and to maintain systemic screening for bone tumors. With the histopathology report of the anatomical specimen, patients are referred to clinical oncology for appropriate treatment (Figures 2A-2C).

## RESULTS

Out of six patients who underwent the proposed surgery, three were women (50%) with an average age of 21 years. Half of the sample had ASA I anesthetic risk and the other half ASA II. The average hospital stay was 7.8 days, and the average operative duration was 3.25 hours (Table 1).

**Table 1:** Clinical profile of patients undergoing surgery.

Patient	Age	Sex	Side	Comorbidities	ASA	Days hospitalized	Operative duration (hours)
1	45	Female	Right	Asthma/COPD	II	6	4
2	11	Female	Left	No	II	23	4
3	25	Male	Right	No	II	7	3.5
4	11	Female	Left	No	I	5	4
5	18	Male	Right	No	I	3	2
6	16	Male	Left	No	I	3	2

COPD: chronic obstructive pulmonary disease.

The lesions varied significantly in size, from 4.2 to 14 cm, with an average of 9.3 cm and different diagnoses (Table 2).

**Table 2:** Profile of lesions and surgical complications.

Patient	Biopsy	Anatomical specimen	Lesion size (cm)	Complications within 30 days	Complications after 30 days
1	Grade II classic central chondrosarcoma	Grade II classic central osteosarcoma	4.2	Fibular paresthesia	Uneventful
2	Classic central osteosarcoma	Grade II classic central osteosarcoma	5.3	Uneventful	Uneventful
3	Giant cell tumor (GCT) with aneurysmal changes	Fibrohistiocytic proliferation corresponding to residual GCT stroma after Denosumab	8	Uneventful	Surgical site infection
4	Epithelioid hemangioma	Kaposiform hemangioendothelioma	12.5	Deep peroneal and saphenous nerve injury; Skin necrosis	Surgical site infection
5	Classic central osteosarcoma	Classic central chondroblastic osteosarcoma	14	Uneventful	Uneventful
6	Telangiectatic osteosarcoma	Classic central osteosarcoma	12	Uneventful	Asymptomatic pseudoarthrosis of the proximal focus

Outpatient follow-up showed neuropraxia of the peroneal nerve within 30 days in two patients who recovered function with specific oral medication. After 30 days, the surgical site became infected in two cases, one of which the patient underwent allograft removal, venous antibiotic therapy, and bone transport. The other patient required a vacuum dressing and intravenous antibiotic therapy to resolve the condition. Consolidation in both foci occurred in three patients within one year of surgery. In two patients, the proximal focus developed pseudoarthrosis, but they remained asymptomatic, and the distal focus consolidated. The last patient required bone transport.

## DISCUSSION

As these pathologies have low incidence in the general population and spread easily to adjacent structures due to their local anatomy, malignant tumors in the ankle are often treated aggressively. The literature shows the absence of an ideal option and controversy. The choice should be based on conditions intrinsic to the patient (age, comorbidities, among others) and the tumor (location, histological type, joint involvement).^10^ The technical difficulty of limb-salvage surgery and the need for a wide resection with free margins mean that transtibial amputation is generally preferred as the primary treatment.^11^

Limb-salvage surgery using allografts from tissue banks is still rarely used. Possible factors include difficulty to access the tissue bank, hospital and orthopedist accredited to perform the transplant, lack of experience with allografts, long consolidation process between the allograft and the host bone or specific orthopedic surgical material.

According to Zhao et al.,^11^ the medical decision on the type of surgery to be performed is guided by the presence or absence of the necessary infrastructure, the lack of a good response to chemotherapy, and the involvement of the neurovascular bundle. Patients should be aware of the risks and benefits of each surgery, since functionally infrapatellar amputation has similar long-term results, but the bodily, psychological, and aesthetic changes of an amputation can be disturbing factors.

The study by Moore, Halpern, and Schwartz,^12^ in line with the study by Fin and Simon, recommend answering four questions: 1) Does survival decrease if limb-salvage surgery is performed? 2) Will the function of the limb be maintained or improved? 3) Are there any psychosocial benefits from the procedure? and 4) What are the immediate and long-term morbidities of limb-salvage surgery?

The bone gap present after surgery to resect the distal third of the tibia (including the articular surface) can be reconstructed using the tibia itself where the tumor was, by freezing or radiation; tibia or fibula allograft with some type of synthesis; autograft using the vascularized or nonvascularized fibula and fixation with a plate and screw; or tibia bone transport.

Moore, Halpern, and Schwartz,^12^ in their 2005 study, showed that six out of nine patients who underwent allograft reconstruction required reoperation due to allograft fracture, infection, pseudoarthrosis at the allograft/host bone interface, among other complications. In a similar study, Balsamo, Malinin, and Temple^13^ reported complications in nine of the 12 patients, with no cases of infection, but three cases of pseudoarthrosis, two of fractures, two developed into arthrosis, and two with delayed healing.

In 2018, Zhao et al.^14^ pointed to complications in six out of 11 patients when using allograft and plate and screw fixation in the group in which the graft was combined with the tibia itself, which had been removed, curetted, and prepared with fibula showed a 14% complication rate. The last group that used the double-strut technique had no complications. In a 2019 article, the same author described the double-strut reconstruction of nine patients with diaphysis of the contralateral fibula, which was fixed with a plate and screws in a specific assembly. One patient had an intraoperative complication with a fracture of the donor’s fibula. Postoperatively, the synthesis material failed in one case and the graft/host bone interface failed to consolidate in another.^11^

Hindiskere, Doddarangappa, and Chinder^15^ showed in their article, which is not specific to the distal portion of the tibia, the complications in 16 of the 41 cases that underwent liquid nitrogen freezing (four cases of skin necrosis, one of intraoperative fracture, one of neuropaxis, and one of superficial infection, six of which required a second surgery).

Borzunov, Balaev, and Subramanyam^16^ reported that the main complication was pin tract infection in nine of the 38 patients. One had neuropraxia, in five cases the olive wire had to be changed/removed due to failure, and in one case the fragments had to be immobilized due to loss of reduction.

The leg anteromedial anatomical region has a thinner subcutaneous layer that, along with the need for resection with oncological margins, makes it difficult to cover the material to be implanted. Another complicating factor is the arrangement of the neurovascular bundles, which pass through leg joints and compartments, requiring careful surgical dissection to avoid damaging them.

## CONCLUSION

Despite the technical difficulty of reconstructing the tibial distal region and the complications involved, patients’ satisfaction at being able to move around in the immediate postoperative period, even with the aid of crutches, and the feeling of having their limb preserved can be a step prior to surgeries that do not preserve the limb, as long as patients receive clear guidance during preoperative outpatient consultations and understand the risks and benefits.
